# The DNA demethylation-regulated SFRP2 dictates the progression of endometriosis via activation of the Wnt/β-catenin signaling pathway

**DOI:** 10.1186/s12860-023-00470-9

**Published:** 2023-03-29

**Authors:** Mei Yang, Lin Li, Xiaojie Huang, Hui Xing, Li Hong, Chunfan Jiang

**Affiliations:** 1 Department of Obstetrics and Gynecology, Xiangyang Central Hospital, Affiliated Hospital of Hubei University of Arts and Science, Xiangyang, 441021 Hubei China; 2 Institute of Maternity disease, Xiangyang Central Hospital, Affiliated Hospital of Hubei University of Arts and Science, Xiangyang, Hubei China; 3 Department of Obstetrics and Gynecology, Renmin Hospital of Wuhan University, 238 Jiefang Road, Wuhan, 430060 China; 4 Department of Pathology, Xiangyang Central Hospital, Affiliated Hospital of Hubei University of Arts and Science, Xiangyang, 441021 Hubei China

**Keywords:** SFRP2, Endometriosis, Wnt pathway, Demethylation

## Abstract

**Background:**

Endometriosis cause decreases in life quality and pelvic pain in reproductive-age women. Methylation abnormalities played a functional role in the progression of endometriosis, this study aimed to explore the mechanisms mediated by abnormal methylation in the development of EMS.

**Materials and methods:**

Next-generation sequencing dataset and methylation profiling dataset were used to screen out the key gene SFRP2. Western bolt, Real-time PCR, Aza-2?deoxycytidine treatment, luciferase reporter assay, Methylation-specific PCR , Bisulfite sequencing PCR and lentivirus infection were carried out to detect the methylation status and signaling pathway with the primary epithelial cells. Transwell assay and wound scratch assay were implemented to observe the differences of migration ability with the intervening with the expression of SFRP2.

**Results:**

To define the role of the DNA methylation-regulated genes in the pathogenesis of EMS, we performed both DNA methylomic and expression analyses of ectopic endometrium and ectopic endometrium epithelial cells(EEECs) and found that SFRP2 is demethylated/upregulated in ectopic endometrium and EEECs. The expression of lentivirus carrying SFRP2 cDNA up-regulates the activity of Wnt signaling and the protein expression of ?-catenin in EEECs. SFRP2 impact on the invasion and migration of ectopic endometrium by modulating the activities of the Wnt/?-catenin signaling pathway. The invasion and migration ability of EEECs were significantly strengthened after demethylation treatment including 5-Aza and the knockdown of DNMT1.

**Conclusion:**

In summary, the increased SFRP2 expression-induced Wnt/?-catenin signaling due to the demethylation of the SFRP2 promoter plays an important role in the pathogenesis of EMS, suggesting that SFRP2 might be a therapeutic target for EMS treatment.

**Supplementary Information:**

The online version contains supplementary material available at 10.1186/s12860-023-00470-9.

## Background

As a common disease, EMS affects about 5-10% of women of reproductive age, which causes decreases in life quality and is accompanied by symptoms such as pelvic pain and affects more than 10% of reproductive-age women[1, 2]. Several classical theories including Mül-Lerianosis, retrograde menstruation, and coelomic metaplasia have been proposed to elucidate the pathogenesis of EMS, but the molecular mechanism is still unknown[3, 4].

Many differences were found in gene expression profiles between EMS samples and the normal endometrium tissue samples. Nowadays, microarray technology has become a mature and stable technology, and during the last decade’s bioinformatics analysis has been widely used to identify general genetic of etiology and pathogenesis in many malignant tumors [7–9], but there have been few reports of EMS.Some scholars found that the epigenetic mechanisms including DNA methylation and histone modification closely related to the expression of estrogen receptors and progesterone receptors in patients with EMS[29]. Other scholars evaluated and compared the methylation pattern of Human Homeobox clusters in normal, eutopic (endometrium in the uterine cavity of the EMS patients), and ectopic endometrial tissues, a conserved pattern of methylation alterations in EMS tissues was observed for most of the investigated genes (56 of 84) which indicating epigenetic changes in EMS[32]. And there are some other researches about aberrant endometrial DNA methylation in EMS[38, 39] [40, 41], so we need to clarify the mechanism of aberrant methylom in EMS.

Wnt signaling is an early event in some tissue carcinogenesis, there is evidence that the Wnt signaling pathway also plays a role in the etiology of EMS[20–22]. Characterized histologically by dense fibrous tissue consisting, EMS is researched by many scholars and it was found that treatment with Wingless mouse mamary umor virus (MMTV) integration site family member 3a (Wnt3a) significantly increased the proliferation and migration of endometrial cells in patients with EMS, and significantly enhanced the expression of fiber marker genes, such as α-smooth muscle actin, type I collagen, connective tissue growth factor and fibulin, which were closely related to the contraction of collagen gel[14, 15]. Some studies focus on the effect of endometrial cells-mediated collagen gel contraction on EMS[16]. After treatment with Wnt3a, the contraction of collagen gel I in the endometrial cells in normal endometrium was increased to a level comparable to that in EMS patients[17–19]. In different diseases, SFRP proteins were reported correlating with the Wnt pathway, and their expression was regulated by methylation[24, 34]. For example, SFRP2 is reported to be closely related to Wnt and regulated by methylation in nasopharyngeal carcinoma[35].

This study aimed to explore whether the wnt signaling pathway are mediated by abnomal methylation in the development of EMS.

## Methods

### Microarray data

Next-generation sequencing dataset (GSE135485) and methylation profiling dataset (GSE47359) were obtained from the GEO database. GSE135485 included 54 EMS samples and 4 normal endometrium tissue samples, based on GPL21290 Illumina Human HiSeq 3000 platform. GSE47359 consisted of 3 EMS samples and 6 normal endometrium tissue samples, based on the GPL8490 Illumina Human Methylation 27 platform.

On data processing and identification of differentially expressed genes (DEGs), R software (ver. 3.6.3, https://www.rproject.org/) were used to identify DEGs and differentially methylated genes(DMGs). The matrix file for GSE135485 was downloaded from https://www.ncbi.nlm.nih.gov/geo/query/acc.cgi?acc=GSE135485 and then gene IDs conversion was conducted with strawberry-Perl-5.30.0.1. The data normalization was done with the limma package and then processed with the edge R package to get DEGs. The cut off value of DEGs was set as ｜log_2_FC｜> 4. P < 0.05 was considered to indicate a statistically significant difference.

### Differential methylation genes (DMGs) identification

The HumanMethylation 27 BeadChip array, covers approximately 27,578 CpG sites at different gene regions, embodying the upstream region of the transcriptional start site, 5′untranslated region, exons, 3′untranslated region. The matrix file for GSE47359 was downloaded from http://ftp.ncbi.nlm.nih.gov/geo/series/GSE47nnn/GSE47359/matrix/.

The Champ package of R was used for the identification of CpG sites and DMGs with the threshold P < 0.05 and ｜log_2_FC｜> 0.2. The Champ package is a highly integrated methylation analysis tool, matching the corresponding DMGs with the most differentially methylated CpG sites. A Venn diagram was used to illustrate the intersection between DEGs and DMGs. As a result, upregulated hypomethylated genes were listed.

### GO term and KEGG pathway enrichment

Online analysis tool DAVID was used to conduct Gene ontology (GO) Enrichment Analysis of DEGs into the Cell Components(CC), Molecular Functions(MF), and Biological Processes(BP). All p values < 0.05 were considered to be statistically significant.

### Patient recruitment

This study was initiated on November 11th, 2019 and terminated on April 20th, 2021. All of the women recruited in this study were being at child-bearing age and underwent laparoscopic surgery at the Department of Gynecology of Xiangyang Central Hospital. Five women with endometriosis were recruited before surgery. All these women had not received GnRH-a agonist or hormones treatment for at least six months and were preoperative diagnosed as an ovarian cyst. They were aged between 24 and 39 years old, mean ± SD (32.12 ± 4.90) years; Each case of endometriosis was staged during the operation according to the revised American Fertility Society classification of endometriosis (rAFS) and subsequently confirmed by histology. Among them, two were in rAFS staging III and the other three were in rAFS staging IV. All these patients were in the secretory phase of the menstrual cycle. Ectopic endometrium from the ovarian cyst of these 5 patients were obtained by laparoscopy.

Five women undergoing tubal ligation for sterilization were recruited as controls. All these five patients were aged between 28 and 40 years old, mean ± SD (34.60 ± 4.38) years. No minimal endometriosis was found in these control subjects and no hormones treatment for at least six months. All these women were in the secretory phase of the menstrual cycle. Normal endometrium were obtained by curettage during tubal ligation operation.

### Cell culture

According to our previous study[36], tissues were washed with sterile Hank’s Balanced Salt Solution(HBSS, phenol-red-free) three times, then minced into pieces of approximately 1 mm^3^ and digested in 10 ml of HBSS containing 10 U/ml DNase I (Sigma) and type IV collagenase (0.03%; Sigma, St. Louis, MO) for 40 min at 37 °C. The supernatant was kept and epithelial cells and stromal cells in it were separated by differential centrifugation [21]. To repurify the endometrial cells, the selective attachment was carried out [22]. The endometrial cells were cultured in phenol-red-free DMEM/Ham’s F12 (Invitrogen, Carlsbad, CA) supplemented with 10% v/v fetal bovine serum (FBS; Invitrogen). Next, they were subjected to differential trypsinization and attachment for further purification. Finally, the primary epithelial cells were plated (2 × 104 cells/ml) in dishes in a culture medium as mentioned above. The detect the phenotypic characterization and ensure the purity of endometrial cell > 95%, the primary epithelial cells were tested by dyeing of vimentin and PCK.

### Western blot

Western blot was performed according to our previous study [36] using primary anti-bodies against human SFRP2 (rabbit polyclonal, #HPA002652, Sigma-Aldrich, Merck, USA), anti-β-catenin (#ab6302, Abcam), DNMT1 (#ab13537, Abcam), and mouse monoclonal anti-β-actin (#A5441, Sigma-Aldrich) antibodies. The intensities of the protein bands were measured using the ImageJ (1.49 v) program.

### 5Aza-2′deoxycytidine (aza) treatment of EEECs

As deoxycytidine analogs, 5-Aza-CdR can be irreversibly mixed into DNA for synthesis, thus reducing the ability of DNA to accept methyl under the action of methyltransferase (DNMT). Meanwhile, 5-Aza-CdR forms a covalent complex with DNA methyltransferase (DNMT), reducing the activity of DNMT. And we want to decrease the methylation rate of the promoter of SFRP2 by using this drug. The EEECs were grown and treated with 1 μm of 5-Aza (Sigma-Aldrich #CAS 2353- 33-5) for 3 days for the inhibition of DNA methyltransferase activity.

### Real-time RT PCR

Total RNA was isolated from EMS tissues and EEECs utilizing the TRIzol reagent (Invitrogen, Shanghai, China), and all cRNA transcripts were generated using a primeScript™ RT kit (Qiagen, Hilden, Journal of Molecular Histology1 Germany). All primers (Sangon Biotechnology, China) were listed as fellows: SFRP2, 5′-TGGGGGAAACGGTCGCACTC-3′, and 5′-GGCCACGAGACCATGAAGGAGG-3′. β-catenin, 5′-AAAGCGGCTGTTAGTCACTGG-3′ and 5′-CGAGTCATTGCATACTGTCCAT-3′. The qPCR was performed in triplicate to determine the relative levels of the target mRNA using SYBR premix Ex Taq™ Green II (Takara) in the CFX96 Touch sequence detection system (Bio-Rad, Hercules, CA, USA). Quantitative real-time PCR was conducted ABI 7500 Real-Time PCR System(Applied Biosystems/Life Tech).

### Luciferase reporter assay

To detect the Wnt/β-catenin activation in EEECs, TOP/FLASH and FOP/FLASH reporter gene system (GenePharma Company, Shanghai) were selected to test the Wnt signaling pathway and the Promega dual-luciferase reporter gene assay system was used to measure the reporter activity. TOP/FOP values were used to represent the result. A higher value of TOP /FOP indicates a stronger Wnt pathway activity.

### Methylation-specific PCR (MSP)

Genomic DNA from 5 ectopic endometrium and 5 normal endometrium was isolated using the DNA Extraction Kit (Sangon Biotech, Shanghai, China). In the 50ul system, DNA (2–5 µg) was denatured by NaOH (final concentration 0.2 mol/L) at 37℃ for 10 Min. Add 30 µL of 10 mmol /L hydroquinone and 40.5% sodium bisulfite to mix well, then incubate for 16 h in the condition of air isolation and out of light. The modified DNA passed by a DNA purification column and then eluted by water. At room temperature, it was modified with NaOH (the final concentration was 0.3 mol/L) for 5 min, and then precipitated with ethanol. Dissolve the DNA in 20µL water, stored at -20℃. Two pairs of specific primers were used to amplify the same nucleotide sequence of the tested gene using methylated primer pairs (M) 5′-GGAGTTTTTCGGAGTTGCGC-3′ and 5′-CTCTTCGCTAAATACGACTCG-3′, or unmethylated primer pairs (U) 5′-GTTGGAGTTTTTTGGAGTTGTGT-3′ and 5′-CTCTCTTCACTAAATACAACTCA-3′. The amplified products were detected by DNA agarose gel electrophoresis and analyzed by gel scanning.

### Bisulfite sequencing PCR

Genomic DNA from 5 ectopic endometrium and 5 normal endometrium was isolated using the DNA Extraction Kit (Sangon Biotech, Shanghai, China). According to the manufacture’s instruction, and bisulfite modification was performed with the EZ DNA Methylation Gold Kit (Tianmo Technology, Beijing, China). Primer(Sangon Biotechnology, China) sequences for bisulfite sequencing were listed as follows: forward(M818-F)5′-TTTATGTTTGGTAATTTAGTAGAAATTT-3′ and reverse (M818-R) 5′-ATTTTACRTTAAAAATACCCCTCAC-3′. This area was 302-bp fragments including 28 CpG dinucleotides. The PCR conditions were: pre-denaturation at 95 °C for 3–5 min, denaturation at 94 °C for 30s, 55–60 °C for 30s, and 72 °C for 30s, 35cycles totally. Then, the sequence containing the SFRP2 sequence was sequenced(Sangon Biotech, Shanghai, China).

### Plasmid construction and lentivirus production

The human SFRP2 lentiviral vectors were purchased from GenePharma and transfected EEECs according to standard manufacturer protocols. Furthermore, lentiviral vectors to knockdown DNMT1 expression were generated by the GenePharma Company, (Shanghai), and the interfering sequence is as follows: DNMT1-Homo-2664 GGAGCTGTTCTTGGTGGATGA. Three kinds of infection sequence were tested in the preliminary experiments, and one is useful as mentioned above. Post-infected cells were cultured for one week consecutively and lentivirus infection condition of target cells were determined by observing the expression time and intensity of GFP. To screen the stably transfection clusters, at the basis of transient infection, puromycin with minimum lethal concentration lasts for at least 4 days.

### Immunohistochemistry

A cohort of 84 formalin-fixation paraffin-embedded specimens (FFPE), including 28 EMS ectopic endometrium, 28 eutopic endometrium and 28 normal endometrium were retrieved from Xiangyang Central Hospital from 2006 to 2020 with necessary clinical information. 28 eutopic endometrium and ectopic endometrium were get from 28 ovarian endometrial cyst patients which were aged between 25 and 43 years old, mean ± SD (35.05 ± 8.70) years; normal endometrium patients were aged between 29 and 48 years old, mean ± SD (41.80 ± 6.22) years. All the cases were reviewed by two senior pathologists separately again to ensure the diagnosis accuracy.

Immunohistochemical staining for SFRP2 was performed with 3-µm-thick sections using the Ventana Benchmark ULTRA automated staining system (Ventana Medical Systems, Tucson, AZ) according to the manufacturer’s protocol. SFRP2 (Abcam), the primary antibodies were added on the cell sections for two hours, Sections were incubated with a secondary antibody and visualized with 3, 3’-diaminobenzidine tetrahydro-chloride (DAB; Golden Bridge, Beijing, China). Sections were then subjected to nuclear counterstaining (blue staining) with hematoxylin. Two investigators were asked to review and score the anti-SFRP2 staining on the stained sections by adding the percentage score with the intensity score. Staining intensity was scored as 0 (negative), 1 (weak), 2 (moderate) and 3 (strong), while staining percentage was scored as 0 (< 10% staining), 1 (11–25% staining), 2 (25–75% staining) and 3 (≥ 75% staining). And these two fractions were added together, score 0–3: low; 4–6: moderate; 7–9: high.

### Transwell assay

BD matrigel and 1640 were diluted in a ratio of 1:3 and 80ul was added to the upper chamber of the transwell chamber(8 μm; Millipore, Billerica, MA). EEECs were treated with 5-Aza, sh-DNMT1 or lentivirus carrying SFRP2-cDNA. Cell suspensions were configured according to the concentration of 200ul of serum-free medium containing 2.5 × 10^4^ cells. 500 µl DMEM medium was added to the subchamber wells of the Transwell plate and the chamber was placed into the plate with care not to produce bubbles. Celcultures were grown in 37℃ incubator containing 5%CO2 for 24 h. Assays were then stopped by removing the non-invading cells in the top chamber with swabs. The chamber was removed and the medium was washed with PBS and the chamber was stained for 10 min; next the crystal violet of the cleaned chamber surface was washed with water, the cells in the upper chamber were wiped with a cotton swab and photographed under an inverted microscope. Cells in five visual fields per insert were counted (400× magnification).

### Wound scratch assay

Log-growth EEECs were digested with trypsin and cells were evenly spread out into 6-well plates according to experimental grouping. They were incubated in an incubator at 5%CO2 and 37℃. When the cells grows to 80 -90% confluence, a straight line was drawn in the well using the appropriate pipette gun head along a sterilization ruler. The shed cells were washed out three times with PBS. In the presence of serum, untreated cells should migrate and fill the scratch area after approximately 48 h. Twenty-four hours after scratching, different treatments displayed remarkable effects on cellular migration in preliminary experiments, so this time point was chosen to end the assay. EEECs were treated with 5-Aza, sh-DNMT1 or lentivirus carrying SFRP2-cDNA as described above. Pictures were taken at 0 and 24 h under an inverted microscope. The relative migration length in five random fields was measured with ImageJ for further quantitative analysis.

### Statistical analysis

All the experiments were repeated at least three times. SPSS 13 software was used for statistical analysis of all experimental data. The data were normally distributed. The comparison between the two groups was estimated by Student’s t-test. A p-value < 0.05 was considered significant. Chi-square was used in the Statistical analysis of immunohistochemistry data.

## Results

### Identification of aberrantly methylated genes

**Fig. 1:**
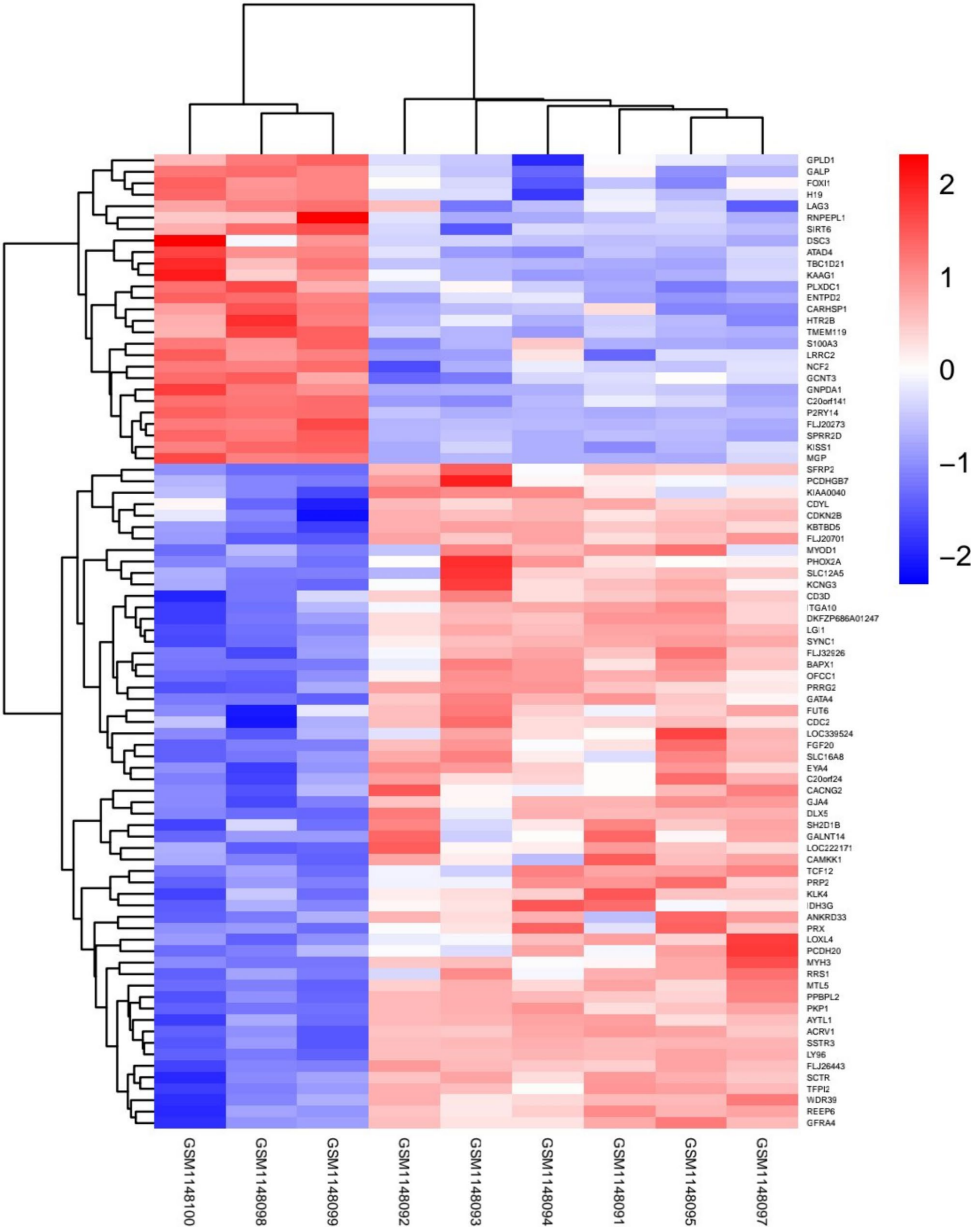
Heatmap clustering of the gene methylation status from GSE47359 in 3 EMS samples vs. 6 normal endometrium tissue samples

Heatmap clustering of the gene methylation status from GSE47359 in 3 EMS samples vs. 6 normal endometrium tissue samples was made. A total of 3215 CpG sites were found and associated with the profile of differentially **methylated** genes from a microarray analysis from the GEO database (Fig. 1). A total of 85 differentially **methylated** genes were identified after the screening, 27 genes were hypermethylated and 58 genes were hypomethylated in EMS. After the GO analysis of the low methylation expression, the functions of these hypomethylated genes were explored in several important cell processes, including repressor, secreted, and signaling (Table 1).

#### Legend

Datas were reported as mean ± SD. All experiments were carried out in three or more replicates, and repeated at least twice. Statistically significant was displayed as *P<0.05, **P<0.01, ***P<0.001.

**Table 1:** GO analysis of the low methylation expression gene from the GSE47359 data

Category	Term	Count	PValue
GOTERM_CC_DIRECT	GO:0000790 ~ nuclear chromatin	4	0.005055271
GOTERM_BP_DIRECT	GO:0042493 ~ response to drug	3	0.011057299
GOTERM_BP_DIRECT	GO:0031668 ~ cellular response to extracellular stimulus	2	0.029690877
GOTERM_MF_DIRECT	GO:0003713 ~ transcription coactivator activity	3	0.030570734
GOTERM_BP_DIRECT	GO:0048646 ~ anatomical structure formation involved in morphogenesis	2	0.031939077
GOTERM_BP_DIRECT	GO:0045944 ~ positive regulation of transcription from RNA polymerase II promoter	5	0.043516788
GOTERM_CC_DIRECT	GO:0005667 ~ transcription factor complex	3	0.053312753
GOTERM_CC_DIRECT	GO:0090575 ~ RNA polymerase II transcription factor complex	2	0.058856003
GOTERM_BP_DIRECT	GO:0050680 ~ negative regulation of epithelial cell proliferation	2	0.099183706
GOTERM_BP_DIRECT	GO:0035914 ~ skeletal muscle cell differentiation	2	0.099183706

After the GO analysis of the low methylation expression, the functions of these hypomethylated genes were explored in several important cell processes, including repressor, secreted, and signaling (Table 1)

### Screening for DEGs

**Fig. 2:**
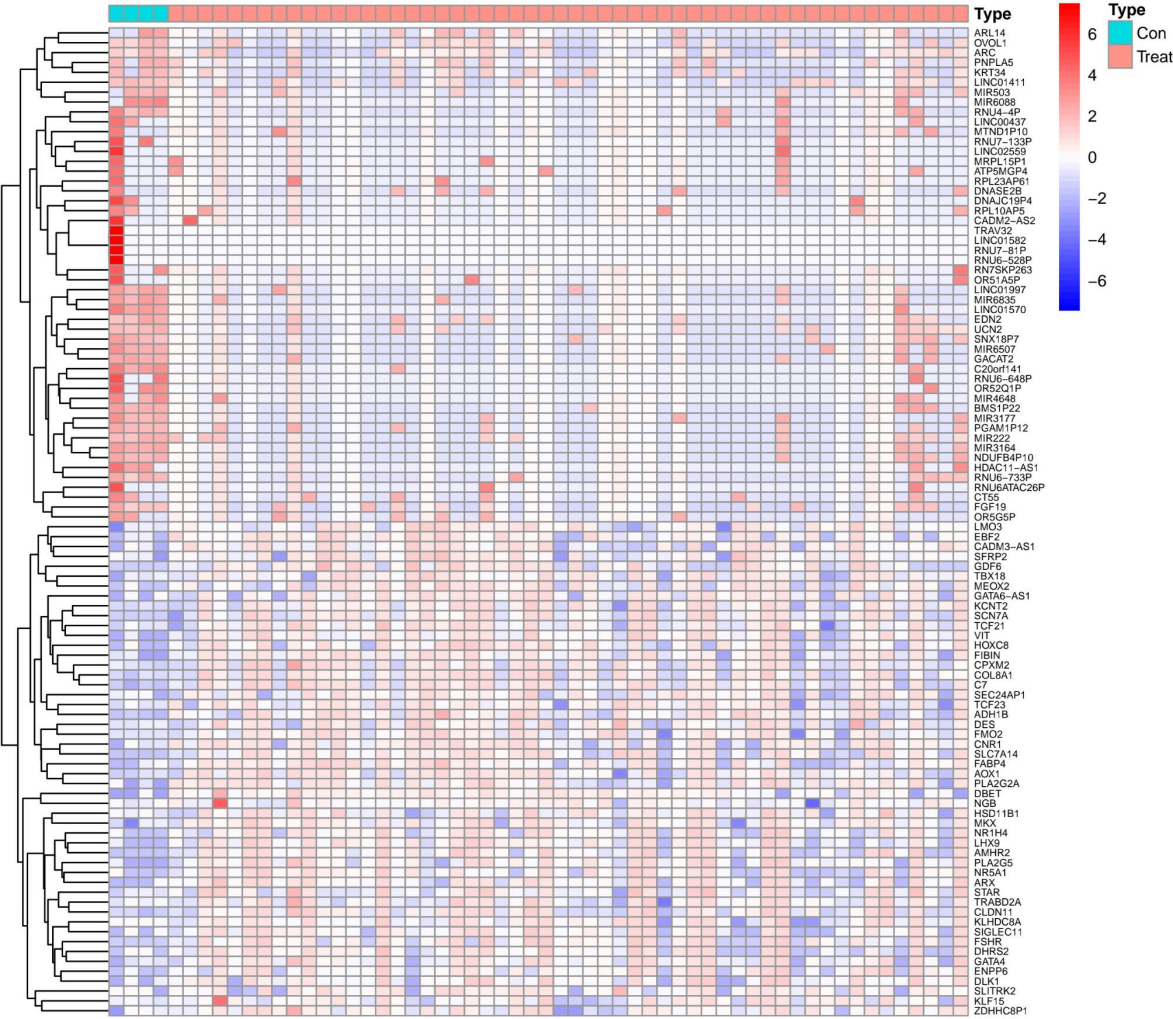
Heatmap clustering of differentially expressed genes in mRNA expression profiling datasets (GSE135485), which includes 54 EMS samples and 4 normal endometrium tissue samples

**Fig. 3:**
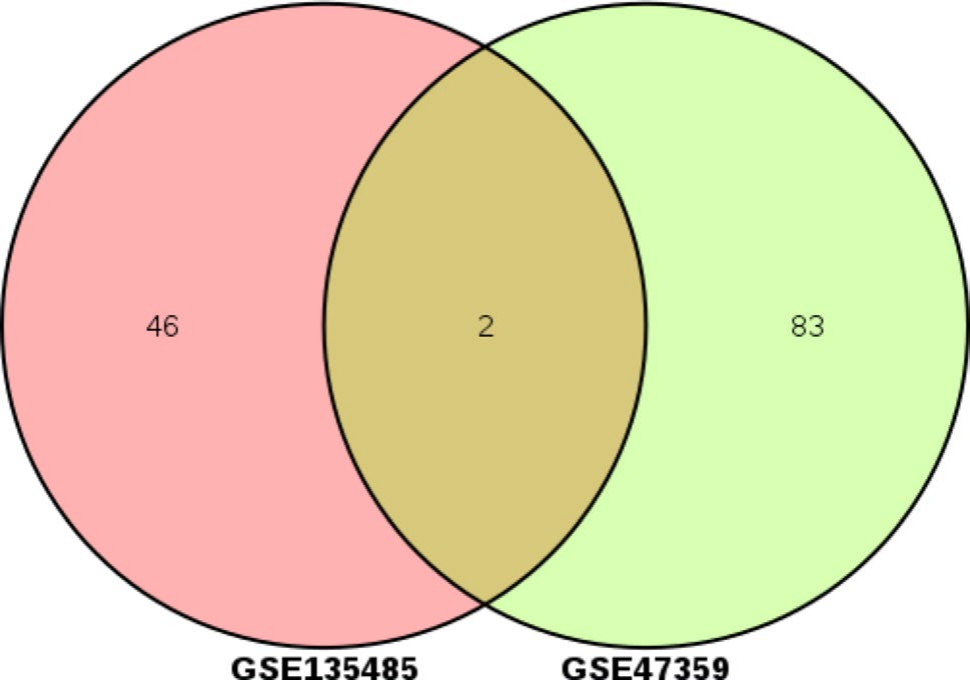
Identification of aberrantly methylated-differentially expressed genes in mRNA expression profiling datasets (GSE135485) and gene methylation profiling datasets (GSE47359).

To identify DEGs in EMS compared to healthy controls, one next-generation sequencing dataset (GSE135485) with the 54 EMS and 4 healthy controls has been analyzed using the linear modeling approach. A total of 134 DEGs were identified after the screening, of which 48 genes were upregulated and 86 were downregulated (｜log_2_FC｜> 4, p < 0.05) (Fig. 2). The downregulation genes were not the research focus, so we did not present them here. 48 upregulation genes (｜log_2_FC｜> 5, p < 0.05) were selected for subsequent bioinformatic analysis. The significant terms of GO enrichment analysis performed by DAVID were illustrated in Table 2. The intersection of hypomethylated genes and overexpressed genes in EMS were get and there are two upregulation and demethylation genes(Fig. 3). Among them, SFRP2 was seldom described in EMS yet. Therefore, we further verified the expression of SFRP2 and study its function in the development of EMS.

**Table 2:** GO analysis of the upregulation genes from the GSE135485 data

Category	Term	Count	PValue
GOTERM_BP_DIRECT	GO:0008584 ~ male gonad development	7	1.35E-07
GOTERM_MF_DIRECT	GO:0000980 ~ RNA polymerase II distal enhancer sequence-specific DNA binding	4	5.23E-04
GOTERM_BP_DIRECT	GO:0008585 ~ female gonad development	3	6.58E-04
GOTERM_MF_DIRECT	GO:0043565 ~ sequence-specific DNA binding	7	0.001463497
GOTERM_MF_DIRECT	GO:0001077 ~ transcriptional activator activity, RNA polymerase II core promoter proximal region sequence-specific binding	5	0.002513506
GOTERM_BP_DIRECT	GO:0006366 ~ transcription from RNA polymerase II promoter	6	0.009651672
GOTERM_BP_DIRECT	GO:0045944 ~ positive regulation of transcription from RNA polymerase II promoter	8	0.01158106
GOTERM_MF_DIRECT	GO:0016491 ~ oxidoreductase activity	4	0.012548298
GOTERM_BP_DIRECT	GO:0007584 ~ response to nutrient	3	0.015396063
GOTERM_BP_DIRECT	GO:0050810 ~ regulation of steroid biosynthetic process	2	0.020307471
GOTERM_MF_DIRECT	GO:0047498 ~ calcium-dependent phospholipase A2 activity	2	0.021652815
GOTERM_BP_DIRECT	GO:0036149 ~ phosphatidylinositol acyl-chain remodeling	2	0.040211948
GOTERM_BP_DIRECT	GO:0036148 ~ phosphatidylglycerol acyl-chain remodeling	2	0.045126018
GOTERM_BP_DIRECT	GO:0036150 ~ phosphatidylserine acyl-chain remodeling	2	0.045126018
GOTERM_BP_DIRECT	GO:0036152 ~ phosphatidylethanolamine acyl-chain remodeling	2	0.059721243
GOTERM_BP_DIRECT	GO:0050482 ~ arachidonic acid secretion	2	0.059721243
GOTERM_MF_DIRECT	GO:0008270 ~ zinc ion binding	7	0.0613717
GOTERM_BP_DIRECT	GO:0036151 ~ phosphatidylcholine acyl-chain remodeling	2	0.066936914
GOTERM_MF_DIRECT	GO:0003682 ~ chromatin binding	4	0.068885928
GOTERM_BP_DIRECT	GO:0009755 ~ hormone-mediated signaling pathway	2	0.069330097
GOTERM_MF_DIRECT	GO:0004623 ~ phospholipase A2 activity	2	0.072674345
GOTERM_MF_DIRECT	GO:0017147 ~ Wnt-protein binding	2	0.072674345
GOTERM_BP_DIRECT	GO:0070374 ~ positive regulation of ERK1 and ERK2 cascade	3	0.073862462
GOTERM_CC_DIRECT	GO:0090575 ~ RNA polymerase II transcription factor complex	2	0.07673751
GOTERM_BP_DIRECT	GO:0050873 ~ brown fat cell differentiation	2	0.078843017
GOTERM_MF_DIRECT	GO:0003700 ~ transcription factor activity, sequence-specific DNA binding	6	0.081625912
GOTERM_MF_DIRECT	GO:0004879 ~ RNA polymerase II transcription factor activity, ligand-activated sequence-specific DNA binding	2	0.083902905
GOTERM_BP_DIRECT	GO:0006654 ~ phosphatidic acid biosynthetic process	2	0.085915314
GOTERM_CC_DIRECT	GO:0005576 ~ extracellular region	8	0.08929768
GOTERM_BP_DIRECT	GO:0035094 ~ response to nicotine	2	0.090600685
GOTERM_BP_DIRECT	GO:0030522 ~ intracellular receptor signaling pathway	2	0.092934565
GOTERM_BP_DIRECT	GO:0010811 ~ positive regulation of cell-substrate adhesion	2	0.092934565
GOTERM_BP_DIRECT	GO:0048468 ~ cell development	2	0.097584789

The significant terms of GO enrichment analysis performed by DAVID from the GSE135485 data

### Increased SFRP2 expression in EMS tissues and EEECs

**Fig. 4:**
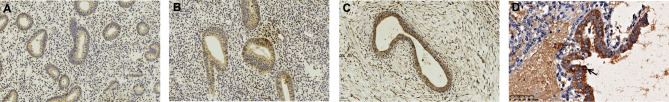
Immunohistochemical staining of SFRP2 protein in different endometrium tissues. **A** Normal endometrium. **B** Eutopic endometrium (endometrium in the uterine cavity of the EMS patients). **C** ectopic endometrium. **D** Through the immunohistochemistry staining, it was found that SFRP2 was clearly located in the cytoplasm and was yellowy-brown

The immunohistochemistry experiments’ results of normal endometrium(Fig. 4A), eutopic endometrium of EMS patients(Fig. 4B) and ectopic endometrium(Fig. 4C) were presented in Fig. 4., Compared with the normal endometrium and eutopic endometrium, the protein expression levels of SFRP2 were significantly increased in ectopic endometrium (χ2 = 17.907, p < 0.001). The SFRP2 expression in the eutopic endometrium of EMS patients showing no differences compared with normal endometrium samples( χ2 = 2.9, 8,p = 0.087 > 0.05). Meanwhile, through the immunohistochemistry experiments, it was found that SFRP2 was located in the cytoplasm of EEECs and was yellowy-brown(Fig. 4D).

**Fig. 5:**
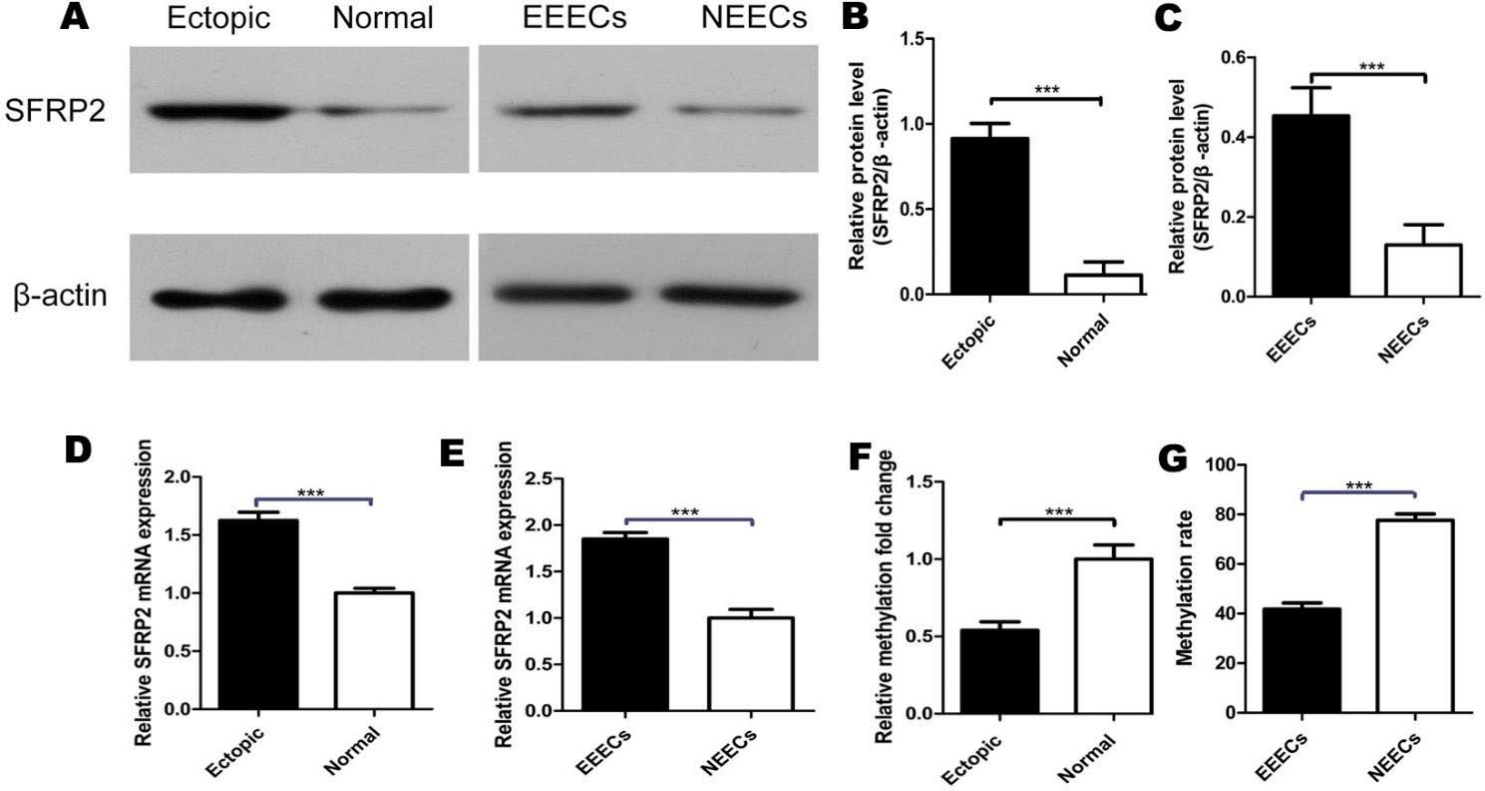
Increased SFRP2 expression in EEECs and endometriosis tissues. **A** Western blot analysis of SFRP2 protein level in ectopic endometrium vs. NE(Normal endometrium) and protein level in EEECs vs. NEECs. Full-length blots/gels are presented in Supplementary Figure 1. **B** Western blot data of SFRP2 protein level in ectopic endometrium vs. NE **C** Western blot data on SFRP2 protein level. in EEECs vs. NEECs. **D** Real-time RT PCR. The mRNA expression levels of SFRP2 in ectopic endometrium vs Normal endometrium. **E** Real-time RT PCR. The mRNA expression levels of SFRP2 in EEECs vs NEECs. **F** MSP. The methylation rates of normal endometrium and ectopic endometrium endmetrium were detected by MSP respectively. **G** Bisulfite sequencing PCR on SFRP2 promoter in EEECs and NEECs.

The SFRP2 protein expression in EMS vs. normal endometrium and EEECs vs. NEECs assessed using western blot were exhibited in Fig. 5A. All the experiments were repeated three times, the results of statistical analysis were shown in Fig. 5B and C. It was found that SFRP2 protein levels were significantly upregulated in ectopic endometrium/EEECs, compared with normal endometrium/NEECs (p < 0.001. Next, through the RT-PCR, it was found that compared with normal endometrium/NEECs, the mRNA expression levels of SFRP2 in ectopic endometrium/EEECs were significantly increased (P < 0.001, Fig. 5D and E).

### Demethylation of the SFRP2 promoter in EMS

From the MSP data, it was found that compared with that in normal endometrium, the SFRP2 promoter region was hypomethylated in EMS, (P < 0.0001, Fig. 5F). To further investigate whether the activation of SFRP2 is related to the methylation status of the promoter, Bisulfite sequencing PCR was used in EEECs and NEECs. Direct sequencing analysis of a 302-bp fragment including 28 CpG dinucleotides in the SFRP2 promoter was performed. Differential methylation was observed in 28 CpG dinucleotides of the promoter in these two kinds of cells. We found that the percentage of methylated CpG dinucleotides in EEECs and NEECs was 41.8% and 77.6%, respectively, P = 0.002 (Fig. 5G), This suggests that hypomethylation of the SFRP2 promoter in EMS.

### SFRP2 was upregulated due to the reduced methylation status of the promoter

**Fig. 6:**
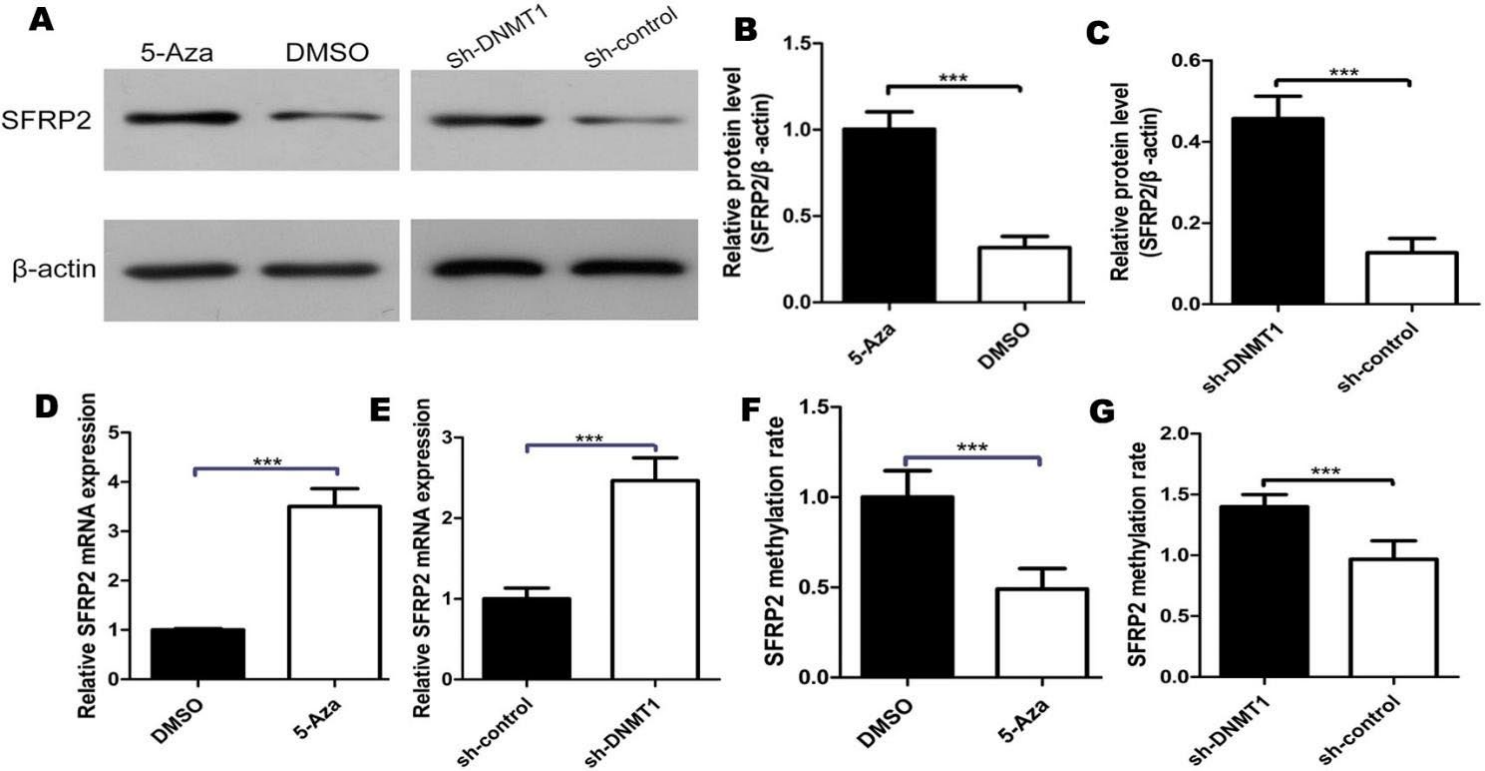
SFRP2 was upregulated due to the reduced methylation status of the promoter. **A** Western blot analysis of SFRP2 protein level in EEECs under treatment of 5-Aza vs DMSO or in EEECs under transfection with DNMT1 shRNAs vs control group. Full-length blots/gels are presented in Supplementary Figure 1. **B** Western blot data of SFRP2 protein level. The protein expression levels of SFRP2 in EEECs treated with 5-Aza vs DMSO group. **C** Western blot data of SFRP2 protein level. The protein expression levels of SFRP2 in EEECs transfected with DNMT1 shRNAs vs control group. **D** Real-time RT PCR. The mRNA expression levels of SFRP2 in EEECs treated with 5-Aza vs control group. **E** Real-time RT PCR. The mRNA expression levels of SFRP2 in EEECs transfected with DNMT1 shRNAs vs control group. **F** MSP. The methylation rate of SFRP2 promoter in EEECs treated with 5-Aza vs DMSO group. **G** MSP. The methylation rate of SFRP2 promoter in EEECs transfected with DNMT1 shRNAs vs control group

DNA methyltransferase(DNMT) is a pivotal isozyme for DNA methylation. To further understand the influence of promoter methylation on SFRP2 expression, the depletion of DNMT was performed in EEECs. There were several DNMT, and through preliminary experiments, it was found that the level of SFRP2 promoter methylation was significantly reduced in EEECs by DNMT1 knockout. So next we choose lentiviral vectors to knockdown DNMT1 expression in the following experiments. To detect the SFRP2 protein expression in EEECs, western-blot was used. After the treatment with 5-Aza-2′-deoxycytidine, it was found that the levels of SFRP2 protein were significantly increased, meanwhile, the levels of SFRP2 protein were significantly increased after knockdown of DNMT1 (Fig. 6A-C, p < 0.001). After the treatment with 5-Aza-2′-deoxycytidine, and the mRNA(p < 0.001) levels of SFRP2 were significantly increased, at the same time, from MSP data, it was found that the level of SFRP2 promoter methylation rate in EEECs was significantly reduced(P < 0.01) (Fig. 6D F) in EEECs. And after knockdown of DNMT1 in EEECs, it was found that the mRNA (P < 0.0001) levels of SFRP2 were significantly increased and the level of SFRP2 promoter methylation rate was significantly reduced(Fig. 6E and G).

### The regulation of the wnt signaling pathway after ectopic expression of SFRP2 in EMS

**Fig. 7:**
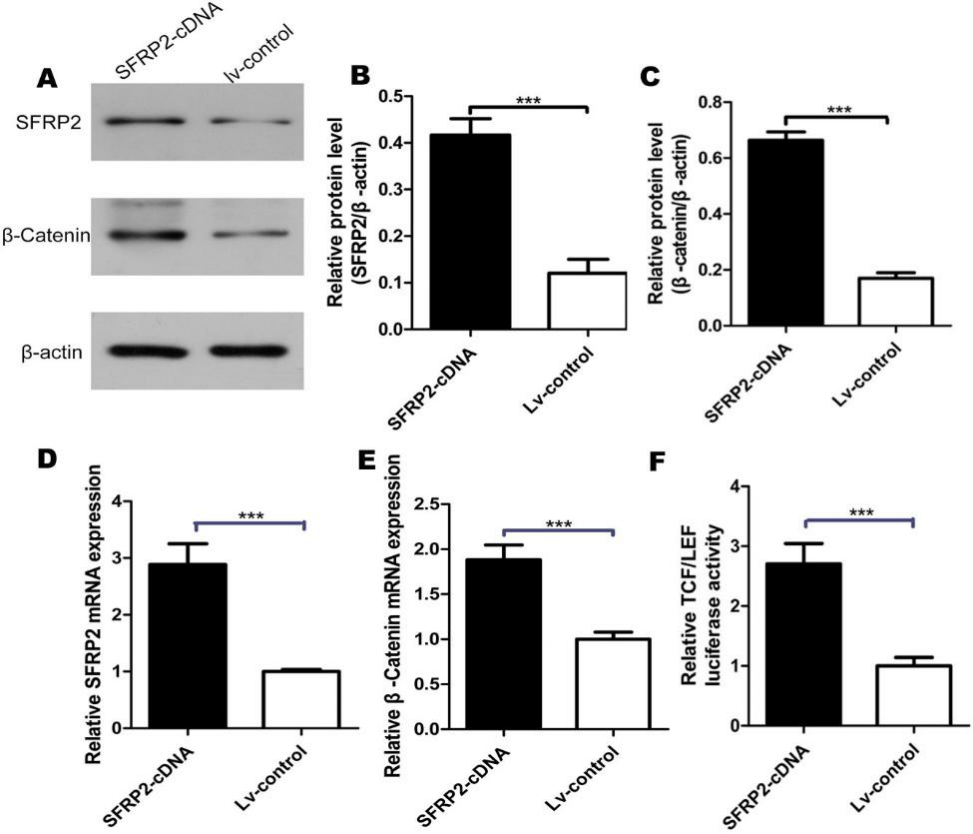
Increase of Wnt signaling gene expression and activity after ectopic SFRP2 expression in EEECs. **A** Western blot analysis. EEECs were transfected with SFRP2 cDNA or lv-control and subjected to Western blot analysis. Full-length blots/gels are presented in Supplementary Figure 1. **B** Western blot data of SFRP2 protein level. The protein expression levels of SFRP2 in EEECs transfected with SFRP2 cDNAvs control group. **C** Western blot data of beta-Catenin protein level. The protein expression levels of beta-Catenin in EEECs transfected with SFRP2 cDNAvs control group. **D** Real-time RT PCR. The mRNA expression levels of SFRP2 in EEECs transfected with SFRP2 cDNA vs control group. **E** Real-time RT PCR. The mRNA expression levels of beta-Catenin in EEECs transfected with SFRP2 cDNA vs control group. **F** Luciferase reporter assay. The relative TCF/LEF luciferase activity in EEECs transfected with SFRP2 cDNA vs control group

After the transfection of SFRP2 cDNA into EEECs, it was found that SFRP2 protein and mRNA levels were upregulated significantly ( Fig. 7A, B, D and p < 0.001). The protein and mRNA (Fig. 7A C, 7E, p < 0.001) expression of β-catenin, the downstream target gene, were increased after SFRP2 cDNA transfection. Meanwhile, from the Luciferase reporter assay, after the transfection of SFRP2 cDNA, the relative TCF/LEF luciferase activity was also increased compared with the control group(p < 0.001, Fig. 7F).

### Demethylation of SFRP2 promoter changed the invasion and migratory ability of EEECs

**Fig. 8:**
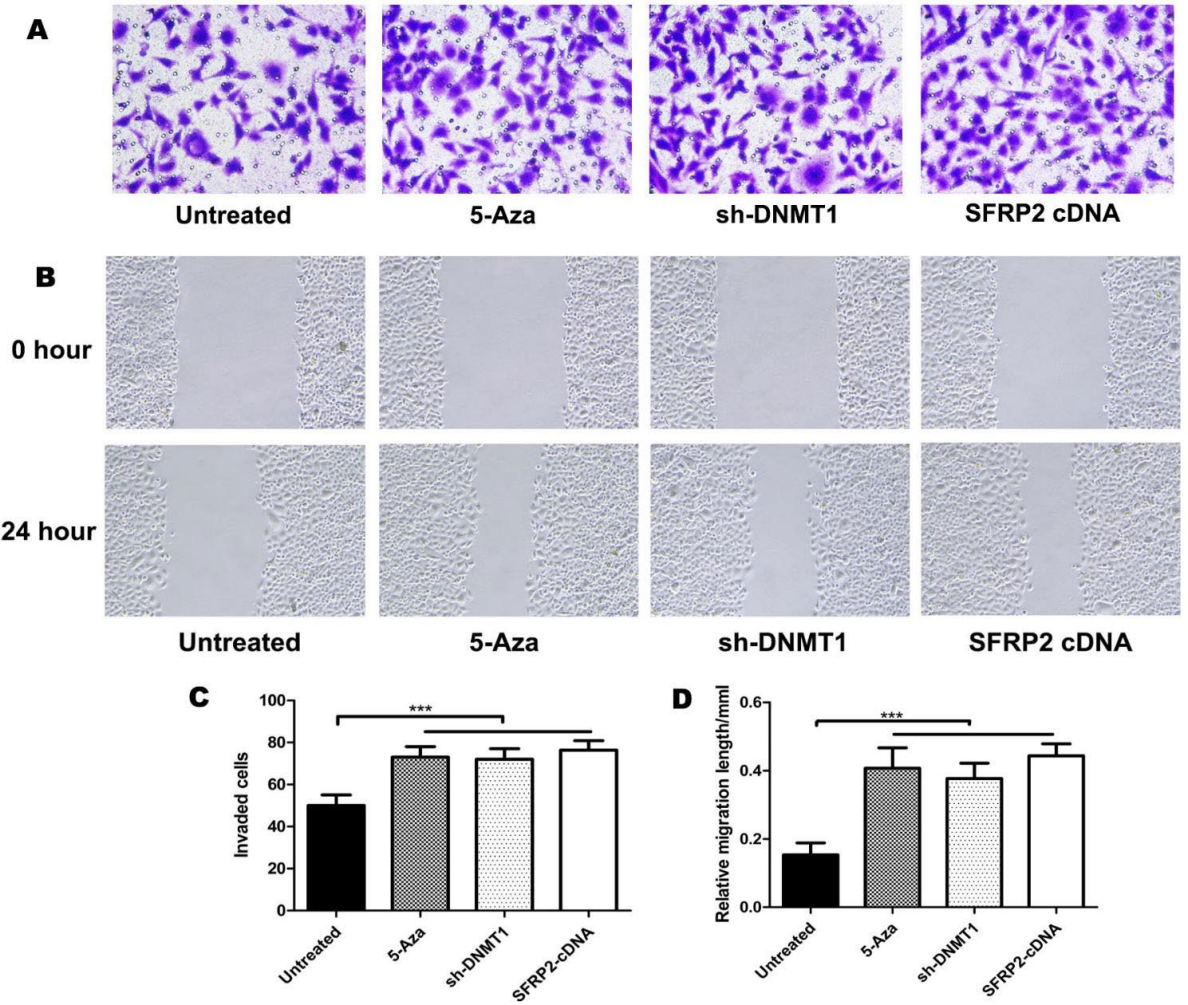
Demethylation of SFRP2 promoter changed the invasion and migratory ability of EEECs. **A** Transwell assays. EEECs were treated by 5-Aza, sh-DNMT1 or lentivirus carrying SFRP2-cDNA respectively and subjected to transwell assays. **B** The numbers of EEECs on the lower surface of the insert membrane of the transwell were counted after each treatment. **C** Wound scratch assays. EEECs were treated with 5-Aza, sh-DNMT1 or lentivirus carrying SFRP2-cDNA respectively and subjected to wound scratch assays. **D** The relative migration length in five random fields was measured after each treatment

To observe the differences in the migration abilities of EEECs influenced by 5-Aza, sh-DNMT1 or lentivirus carrying SFRP2-cDNA intervention, we performed transwell and wound scratch assays. For EEECs, the numbers of cells on the lower surface of the insert membrane of the transwell were counted after each treatment including 5-Aza, sh-DNMT1 or lentivirus carrying SFRP2-cDNA(Fig. 8). All these three intervention were clearly strong impetus promoting the invasion ability of EEECs. Similarly, EEECs had the stronger ability to migrate after 5-Aza, sh-DNMT1 or lentivirus carrying SFRP2-cDNA intervention and left smaller unfilled scratch area (Fig. 8). Significant differences were observed between the untreated cells and any of those three groups receiving different treatment(p < 0.001).

## Discussion

Because of the complexity of biological traits and high heterogeneity, inadequate knowledge about mechanisms at the molecular and cellular levels [11, 37], though many differences were found in gene expression profiles between EMS samples and the normal endometrium tissue samples[4–6]. [10–13], the pathogenesis of EMS has yet to be fully elucidated.

Being considered as a heritable change in gene expression, epigenetics covers abnormal DNA methylation[26], abnormal non-coding RNA, Histone modification changes after translation, etc., among which abnormal DNA methylation is most widely studied[27, 28]. Being as heritable changes in gene expression, epigenetics especially methylation of key regulators plays a critical role in carcinogenesis without alteration in DNA sequence. For example, effects of certain genes with aberrant DNA methylation on HCC and mammary stem cells have been extensively reported[26]. Aberrant DNA methylation could influence some tumor suppressor genes which were key genes involved in the carcinogenesis of HCC and mammary stem cells. Epigenetic modifications were reported to play a role in the pathogenesis of EMS in recent years[29–33], such as epigenetics of estrogen and progesterone receptors and DNA methylation analysis of HOX genes, however, the regulatory mechanism is still unclear. Interestingly, in this study, bioinformatics analysis of DMG microarray and related experiments showed that SFRP2 was significantly demethylated in EMS. From the MSP data, it was found that compared with that in normal endometrium, the SFRP2 promoter region was hypomethylated in EMS. Furthermore, direct sequencing analysis of a 302-bp fragment including 28 CpG dinucleotides in the SFRP2 promoter was performed in EEECs and NEECs. Significant difference was observed in the methylation rate of 28 CpG dinucleotides of the promoter in these two kinds of cells. This suggests that hypomethylation of the SFRP2 promoter in EMS. Methylation of SFRPs was frequently detected in cancers, such as nasopharyngeal carcinoma[35], however, the methylation status of SFRPs has not been reported in EMS yet.

SFRP2 is a member of the various secreted frizzled-related protein (SFRP) family proteins, which are main regulator proteins members of the Wnt pathway. And in different tissues, it could have the opposite activity. Studies have shown that SFRP2 can act as an agonist or antagonist for Wnt signaling[23, 24]. Scholars found that secreted frizzled-related proteins (SFRPs) and some other secreted proteins can competitively displace certain WNT ligands in some cancer models, and increase in SFRP levels attenuates cancer growth, particularly in breast cancer cells[20, 42]. But in the researches of prostate cancer cells in vitro, the overexpression of SFRP1 promotes the growth of BPH1, whereas over-expression of SFRP4 or SFRP3 decreases the proliferation of human PC3 cells[42].

Only two studies concern the experession of SFRPs in EMS. Heinosalo et al. found that after SFRP2 knockout, cell proliferation, and β-catenin protein expression in primary cultured cells with EMS significantly reduced, suggesting that in EMS, SFRP2 acts as an agonist for the Wnt signaling pathway and stimulates lesion growth[24, 25]. The scholars found the increased SFRP2 expression in the EMS lesion, too. Meanwhile, they also found β-catenin and SFRP2 showed similar expression patterns, suggesting that overexpression of SFRP2 promotes the activity of Wnt signal and the growth of EMS lesions[24]. In our study, it was found that compared with the normal endometrium/NEECs, the protein expression levels of SFRP2 were significantly increased in ectopic endometrium and EEECs. And in our research, after the up-regulation of SFRP2 caused by the lentivirus, the up-regulation of the protein expression of β-catenin and activity of Wnt signaling in EEECs were observed, further confirming that SFRP2 may be an important factor in the up-regulation of Wnt signaling in EMS tissues. Our conclusions are consistent with other scholars[24].

The classical Wnt signaling pathway requires β-catenin to enter the nucleus and then bind to the transcription factor TCF/LEF to form a complex, which initiates the transcription of downstream regulatory genes. Scholars found that in the proliferative progenitor cells of colon crypts, the activation of a specific subset of the TCF/LEF family regulate the expression of many target genes that are normally associated with tumorigenesis[42]. Some researches have found that the aberrant activation of Wnt/β-catenin signaling significantly correlated with the pathophysiology of EMS. Some studies found that being a subunit of the cell surface cadherin protein complex, β-catenin act as an intercellular signal transducer in the Wnt signaling pathway and involve in the progress of EMS[20]. Other scholars found that under the regulation of E2, the promotion of MMP9 by Wnt signaling pathway may contribute to the metastasis, detachment, invasion, and implantation of EMS[21]. And there are still researches found that defective endometrial stromal fibroblasts (EMSFs) contribute to EMS, but before implantation, the activation of β-catenin was essential for the key differentiation step of EMSFs[22]. All these studies indicates that WNT signaling in EMS cannot be targeted using the same strategy of cancer, increasingly detailed understanding of WNT signaling in EMS will help us to make clinical decision. In our study, to determine how SFRP2 regulated the Wnt/β-catenin signaling pathway, the expression of downstream target were detected after using transfection of SFRP2-cDNA on EEECs We found that after the up-regulation of SFRP2 caused by the lentivirus, the up-regulation of the protein expression of β-catenin and activity of Wnt signaling in EEECs were observed, further confirming that SFRP2 may be an important factor in the up-regulation of Wnt signaling in EMS tissues. To confirm that the migration of EEECs could be affected by the regulation of the demethylation of SFRP2 promoter, we performed Transwell and wound scratch assays with different treatments. We found that the number of cells on the lower surface of the membrane and the scratch area left unfilled varied significantly after 5-Aza, sh-DNMT1 or lentivirus carrying SFRP2-cDNA intervention. Our data suggest either demethylation of SFRP2 promoter or upregulation of SFRP2 intervention could significantly promote the invasion and migration of EEECs.

There are great clinical relevance in our study. Abnormal activation of the Wnt/β-catenin signaling pathway may be involved in the aggressive phenotype of EMS cells[23]. Pain is a major clinical problem in patients with EMS. Wnt3a and β-catenin are upregulated in various mouse pain models, activating Wnt signaling and possibly contributing to central spinal cord conduction[37]. However, only one literature has reported the regulatory effect of SFRP on the Wnt pathway in EMS, and no literature has been found about the role of methylation on the SFRP expression in EMS. Therefore, the study of SFRP2 in EMS can provide more profound information for the development of EMS and provide new strategies for the clinical control of EMS in the future.

There are obvious strengths in the present study. To date, bioinformatics analysis was rarely used in EMS and there have been few reports regarding the role of SFRP2 in the development of EMS. In this study, next-generation sequencing dataset and methylation profiling dataset were used together and differentially expressed and abnormally methylated genes were found in EMS. Further more, in this study, primary endometrial cells were isolated and cultured, and cell models of transfection were build. This is the first study which clarified the mechanism of SFRP2 demethylation and its interaction with Wnt pathway in the pathogenesis of EMS.

However, there are limitations in the present study: One is that the microarray data were not generated by the authors but from the GEO database. The second limitation of the study is the sample size was relatively small.

## Conclusions

In summary, the increased SFRP2 expression-induced Wnt/β-catenin signaling due to the demethylation of the SFRP2 promoter plays an important role in the pathogenesis of EMS, suggesting that SFRP2 might be a novel regulatory gene and therapeutic target for EMS treatment. This study confirmed that SFRP2 is activated in EMS due to promoter demethylation. Our study could provide new clues to the underlying biological mechanisms.

## Electronic supplementary material

Below is the link to the electronic supplementary material.

Supplementary Material 1

## Data Availability

All data generated or analysed during this study are included in this published article.

## Abbreviations

EMS: endometriosis
EEECs: ectopic endometrium epithelial cells
NEECs: normal endometrial epithelial cells
DEGs: differentially expressed genes
SFRP2: secreted frizzled-related protein 2
DMGs: Differential methylation genes
MSP: Methylation-specific PCR
DNMT: DNA methyltransferase
