# Supplementary material for: The DNA demethylation-regulated SFRP2 dictates the progression of endometriosis via activation of the Wnt/β-catenin signaling pathway
Source: BMC Mol Cell Biol. 2023 Mar 29;24:12. doi: 10.1186/s12860-023-00470-9 (PMC10053136; doi:10.1186/s12860-023-00470-9)

Supplementary Figure 1

The primary endometrial epithelial cells was cultured and verified by immunofluorescence. The primary endometrial epithelial cells were CK19 positive and Vimentin negative.


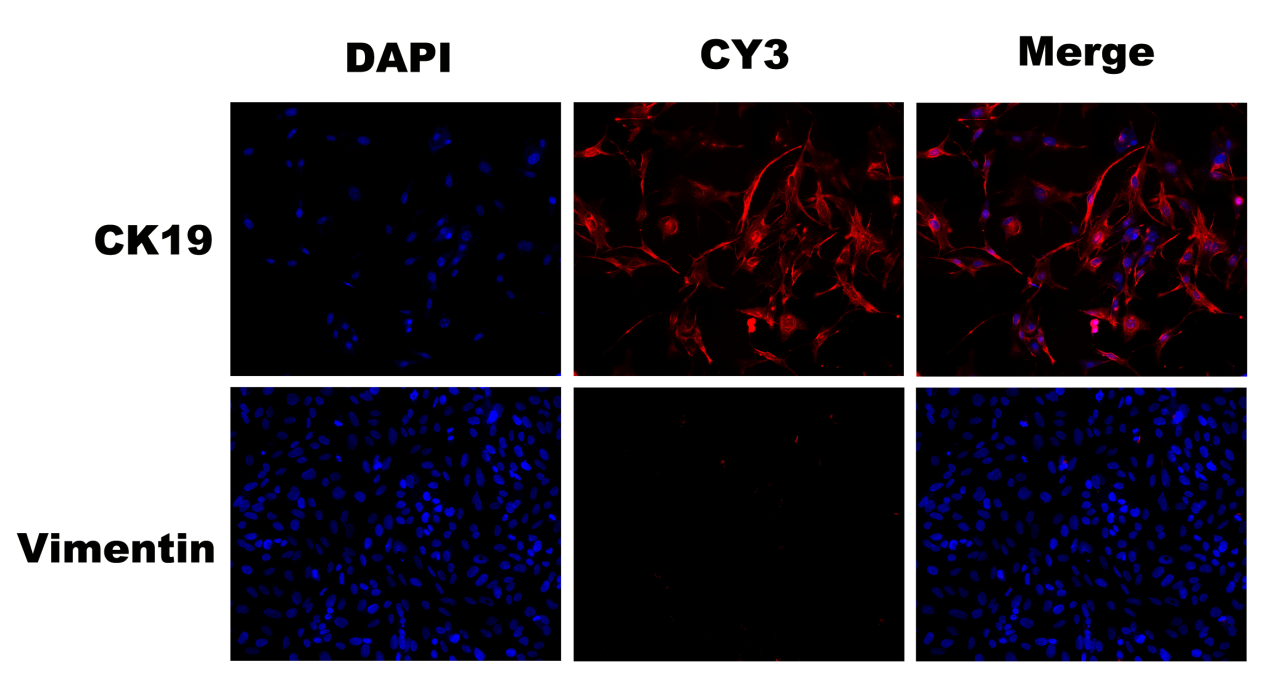


Supplementary Figure 2

The Full-length blots/gels of Western blots.


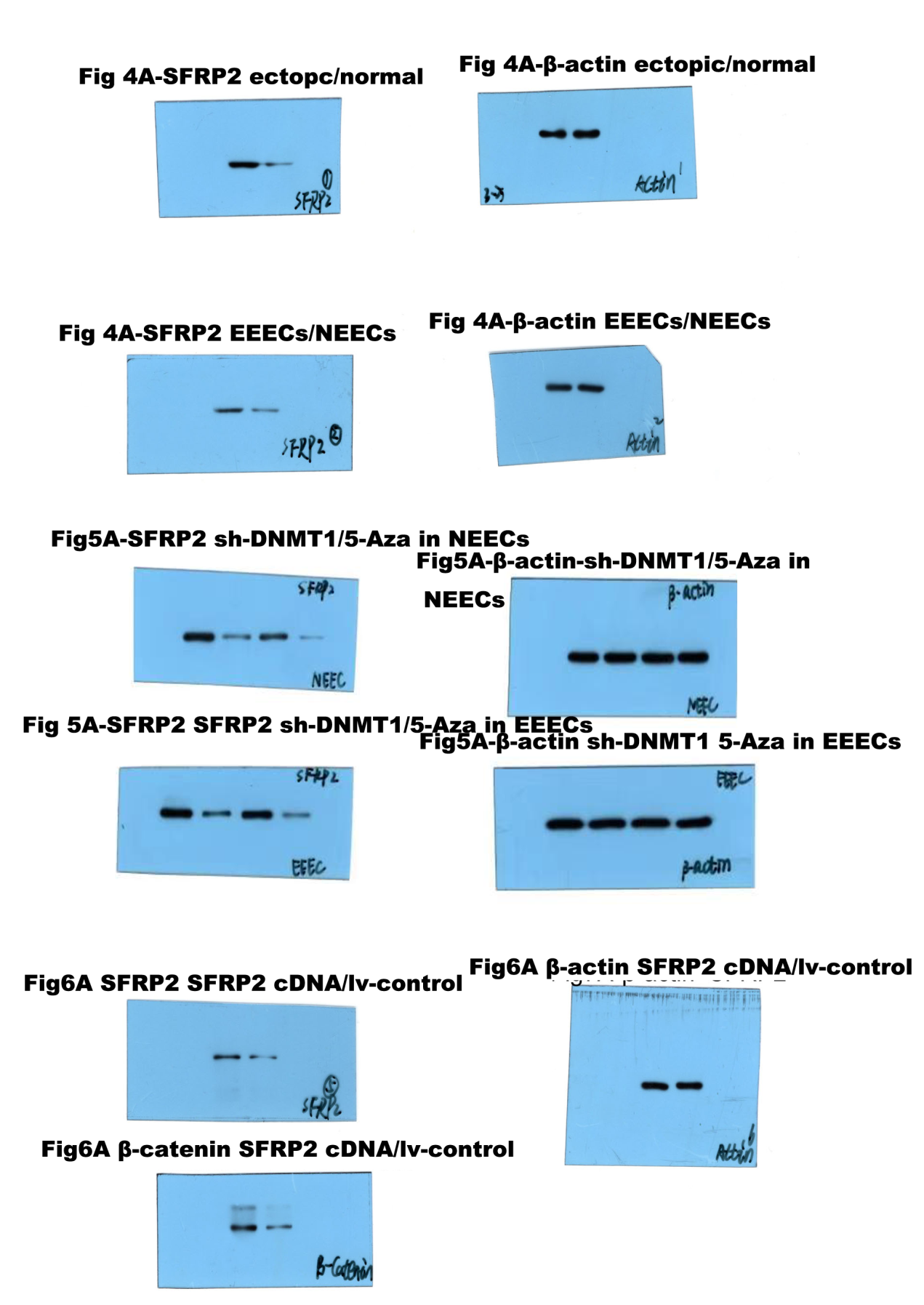

Supplement: Supplementary file 1 — Supplementary Material 1 [file 12860_2023_470_MOESM1_ESM.docx]
